# Species Richness and Evidence of Random Patterns in Assemblages of South American Titanosauria during the Late Cretaceous (Campanian–Maastrichtian)

**DOI:** 10.1371/journal.pone.0108307

**Published:** 2014-09-23

**Authors:** Washington Luiz Silva Vieira, Kleber Silva Vieira, Rômulo Pantoja Nóbrega, Paulo Fernandes Guedes Pereira Montenegro, Gentil Alves Pereira Filho, Gindomar Gomes Santana, Rômulo Romeu Nóbrega Alves, Waltécio Oliveira Almeida, Alexandre Vasconcellos

**Affiliations:** 1 Laboratório de Ecofisiologia Animal, Departamento de Sistemática e Ecologia, Universidade Federal da Paraíba, João Pessoa, PB, Brazil; 2 Museu de Zoologia da Universidade de São Paulo, Ipiranga, São Paulo, SP, Brazil; 3 Programa de Pós-Graduação em Ecologia e Conservação (PPGEC)/Departamento de Biologia, Universidade Estadual da Paraíba, Campina Grande, Paraíba, Brazil; 4 Departamento de Biologia, Universidade Estadual da Paraíba, Campina Grande, Paraíba, Brazil; 5 Departamento de Química Biológica, Centro de Ciências Biológicas e da Saúde, Universidade Regional do Cariri – URCA, Campus do Pimenta, Crato, CE, Brazil; 6 Departamento de Sistemática e Ecologia, Universidade Federal da Paraíba, João Pessoa, PB, Brazil; College of the Holy Cross, United States of America

## Abstract

The Titanosauria were much diversified during the Late Cretaceous, but paleobiological information concerning these sauropods continues to be scarce and no studies have been conducted utilizing modern methods of community analysis to infer possible structural patterns of extinct assemblages. The present study sought to estimate species richness and to investigate the existence of structures in assemblages of the South American Titanosauria during the Late Cretaceous. Estimates of species richness were made utilizing a nonparametric estimator and null models of species co-occurrences and overlapping body sizes were applied to determine the occurrence of structuring in this assemblages. The high estimate of species richness (n = 57) may have been influenced by ecological processes associated with extinction events of sauropod groups and with the structures of the habitats that provided abundant support to the maintenance of large numbers of species. The pseudocommunity analysis did not differ from that expected by chance, indicating the lack of structure in these assemblages. It is possible that these processes originated from phylogenetic inertia, associated with the occurrence of stabilized selection. Additionally, stochastic extinction events and historical factors may also have influenced the formation of the titanosaurian assemblages, in detriment to ecological factors during the Late Cretaceous. However, diagenetic and biostratinomic processes, influenced by the nature of the sedimentary paleoenvironment, could have rendered a random arrangement that would make assemblage structure undetectable.

## Introduction

Sauropods constituted a group of Saurischian dinosaurs that were highly diversified, attaining large dimensions and wide geographic distributions [1]. These herbivorous giants appeared in the fossil record during the Late Triassic and persisted up until the Late Cretaceous [2]–[3]. These dinosaurs probably had a common ancestor related to the basal sauropodomorphs, or “prosauropods” – a globally widespread paraphyletic group of dinosaurs from the Late Triassic and Early Jurassic [2]–[5].

Titanosauria constituted the most diverse sauropod lineage, represented by more than 30 known genera, widely distributed on nearly all continental landmasses during the Late Cretaceous [6]–[7]. However, it is in South America where titanosaurs remains are more common and most diverse in terms of species richness in relation to the other continents [7]. Its diversification and radiation were probably influenced by the global extinction of diplodocoid sauropods in the Late Coniacian during the fragmentation of Gondwana [8]–[12]. Included in this radiation were, for example, saltasaurids, nemegtosaurids, and related forms, such as the genera *Isisaurus* and *Diamantinasaurus* [8]–[10].

Studies of Late Cretaceous South American Titanosauria have dealt predominantly with taxonomic and biochronological aspects, paleogeographic distributions, strategies of locomotion and behavior, reproductive and developmental biology, appendicular morphology, cranial morphology and phylogenetic systematics [4], [7], [9], [13]–[20]. Research on the paleoecology of Titanosauria has been scarce and no studies have been carried out utilizing modern methods of ecological analysis (with estimators of species richness and null models, based on a pseudocommunity analysis) to infer the occurrence of structural patterns in assemblages of South American sauropods or other groups of extinct vertebrates. In general, ecological considerations such as species richness, morphological patterns, strategies of resource utilization, distributions over time and space, and historical and biogeographical factors that could structure species assemblages in a given area have not been examined [21]. The size and the overlapp of ecological niches among sympatric and syntopic species can play important roles in this structuring [22]–[24].

The structure of a vertebrate assemblage can be defined as nonrandom patterns in resource utilization among individuals that coexist in time and space [25]–[26], with species composition and richness having important roles in this structuring [27]. The co-occurrence of vertebrate species in time and space will therefore be determined and structured by their interspecific relationships [28].

Within this context, studies have shown that co-occurrence patterns are commonly attributed to competitive interactions or to environmental filters, and these patterns can be generated by historical factors, habitat associations, and/or species dispersal limits [29]–[31]. The development of null models using pseudocommunities generated by randomizations of the original information by Monte Carlo simulations has provided an important tool for studying biological assemblage structures [32]–[36].

Considering the hypothesis that species co-occurrence in time and space will be determined and structured by their interspecific relationships, the present work sought to estimate the species richness of the South American Titanosauria during the Campanian and Maastrichtian ages (83.5–65 Mya) and to investigate the occurrence of structuring in this assemblage with respect to species co-occurrences and overlapping body sizes.

## Materials and Methods

The list of sauropods presented in this work (Table S1) was prepared based on records of species that coexisted geographically and stratigraphically in the South American fossiliferous formations of the Late Cretaceous (Lower Campanian to the Upper Maastrichtian) as described in the literature ([1]–[9], [37]–[41] and see references in Table S1) and in the databanks of the website Paleobiology Database [42]. We chose to utilize only those records identified to the species level, excluding citations of supraspecific taxonomic groups – thus constructing an assemblage of sympatric and presumably competitive species for analysis. The body size estimates of the different sauropod species (Table S1) were obtained from the literature [1]–[3], [5]–[8], [42]–[46].

The nonparametric estimator Chao1 was utilized to estimate sauropod species richness based on abundance data: S_Chao1_ = Sobs+F^2^_1_/2F_2_, where *Sobs* is the number of species recorded in the assemblage sampled, F_1_ is the number of species represented by only one individual (“singletons”), and F_2_ is the number of species represented by two individuals (“doubletons”) [47]–[49]. The values obtained from the richness estimator, based on 1,000 randomizations without replacement, were plotted indicating the estimated species richness (with 95% confidence intervals) that probably exists in the stratigraphic formations of the Lower Campanian to the Upper Maastrichtian (83.5–65 Mya). The analyses were performed using Estimates 7.5 software [49].

The EcoSim Module of co-occurrence was utilized to test the occurrence of nonrandom patterns of co-occurrence of the Titanosauria species recorded in the stratigraphic formations corresponding to the Lower Campanian to the Upper Maastrichtian in South America [27], [50]. The data for this analysis consisted of a presence (1) absence (0) matrix in which each species corresponded to a line and each stratigraphic formation a column (Table 1). The presence/absence data of the matrix were randomized to produce patterns that would be expected in the absence of competitive interactions between the species. The following EcoSim options were utilized: *C-score* index [50]–[52] as a quantitative co-occurrence index, fixed row and column totals and column constraints, and algorithms of the “Sequential Swap” matrix randomization, with 10,000 randomizations. *C-scores* measure the mean numbers of units in a single block (checkerboard units – CU) for all pairs of species [27], [36], [51]–[52].

**Table 1:** Matrix of occurrence of Titanosauria species recorded in the stratigraphic formations of the Late Cretaceous.

Species/Formation	A	B	C	D	E	F	G	H	I	J	K	L	M	N
*Adamantisaurus mezzalirai*	1	0	0	0	0	0	0	0	0	0	0	0	0	0
*Aeolosaurus colhuehuapiensis*	0	0	0	0	0	1	0	0	0	0	0	0	0	0
*Aeolosaurus rionegrinus*	0	0	1	0	1	0	0	0	0	0	0	0	0	0
*Antarctosaurus wichmannianus*	1	1	0	0	0	0	0	1	1	0	0	0	0	0
*Argyrosaurus superbus*	0	0	0	0	0	1	0	0	0	0	0	0	0	0
*Atacamatitan chilensis*	0	0	0	0	0	0	0	0	0	0	0	0	0	1
*Barrosasaurus casamiquelai*	0	1	0	0	0	0	0	0	0	0	0	0	0	0
*Baurutitan britoi*	0	0	0	0	0	0	1	0	0	0	0	0	0	0
*Bonitasaura salgadoi*	0	0	0	0	0	0	0	0	0	0	1	0	0	0
*Bonatitan reigi*	0	0	0	1	0	0	0	0	0	0	0	0	0	0
*Gondwanatitan faustoi*	1	0	0	0	0	0	0	0	0	1	0	0	0	0
*Laplatasaurus araukanicus*	0	1	0	1	0	0	0	1	0	0	0	0	0	0
*Maxakalisaurus topai*	1	0	0	0	0	0	0	0	0	0	0	0	0	0
*Narambuenatitan palomoi*	0	1	0	0	0	0	0	0	0	0	0	0	0	0
*Neuquensaurus australis*	0	1	0	1	0	0	0	1	0	0	1	0	0	0
*Panamericansaurus schroederi*	0	0	0	1	0	0	0	0	0	0	0	0	0	0
*Pellegrinisaurus powelli*	0	1	0	0	0	0	0	0	0	0	0	0	0	0
*Pitekunsaurus macayai*	0	1	0	0	0	0	0	0	0	0	0	0	0	0
*Puertasaurus reuili*	0	0	0	0	0	0	0	0	0	0	0	1	0	0
*Rocasaurus muniozi*	0	0	0	1	0	0	0	0	0	0	0	0	0	0
*Saltasaurus loricatus*	0	0	0	0	0	0	0	0	0	0	0	0	1	0
*Trigonosaurus pricei*	0	0	0	0	0	0	1	0	0	0	0	0	0	0
*Uberabatitan ribeiroi*	0	0	0	0	0	0	1	0	0	0	0	0	0	0
**Total species richness**	4	7	1	5	1	2	3	3	1	1	2	1	1	1

A: Adamantina Formation, B: Anacleto Formation, C: Angostura Colorada Formation, D: Allen Formation, E: Los Alamitos Formation, F: Bajo Barreal Formation, G: Marília Formation, H: Palacio Formation, I: Plottier Formation, J: Cambambe Formation, K: Bajo de la Carpa Formation, L: Pari Aike Formation; M: Lecho Formation, N: Tolar Formation.

In a structured assemblage, the mean numbers of units in a single block should be significantly higher than the score expected by chance, according to a null model [51]–[52]. The number of units in a single block for any pair of species is calculated by: *CU* = (r_i_–S) (r_j_–S), where r_i_ and r_j_ correspond to the totals in a row, and S is the number of sites occupied by both species. The utilization of fixed row and column totals and column restrictions generate null matrices with the same number of occurrences of sites per species (row totals) and the same number of species per stratigraphic formation (column totals) as observed in the original data. The algorithm of sequential change repeatedly rearranges the original matrix, changing the sub-matrices that preserve the row and column totals, and is not very inclined toward type I or type II errors [27], [36]. The stratigraphic formations utilized for this analysis of co-occurrence are found in Table 1 and Table S1.

The EcoSim Module of Size Overlap was utilized to determine the presence of nonrandom patterns of body size overlapping among species [27]. In this analysis, the estimated total size utilized (log_10_ transformed) for each species was derived from data available in literature [1]–[3], [5]–[9], [42]–[46]. The original matrix was then reformulated to produce random patterns that would be expected in the absence of competitive interactions. The following options were utilized in EcoSim: Variance in segment length as a size-overlap metric, logarithmic transformation, no rounding, and all species in the matrix included in the source pool, with the colonization weights set to 1. Because of the occurrence of variations in segment length, minimum segment length values were utilized.

Segment length was calculated by the ordination of size estimates of the different species. These values represent the differences in body size between two consecutive species. Utilizing the variance in segment length as the size overlap metric, the overall tendency for the observations can be measured. A structured assemblage would have an observed variance significantly smaller than that seen in random assemblages (pseudocommunities). When the minimum segment length values (in meters) are selected, the smallest segment of the assemblage can be calculated by measuring the difference between the closest pair of species. This measure determines whether a minimum space between species is necessary for their coexistence in an assemblage. Thus, in a structured assemblage, the minimum segment length should be significantly greater than that expected by chance [27], [36], [53].

## Results

**Figure 1:**
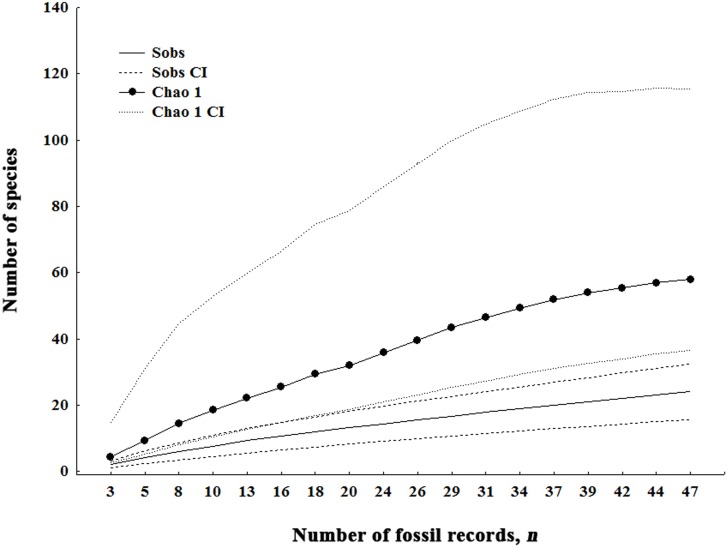
Estimates of species richness represented by rarefaction curves calculate with data of fossil records. The curves were generated from 1000 randomizations with replacement and the sampling units corresponding to the total of fossil record (*n*) in the stratigraphic formations of Late Cretaceous in South America (Campanian–Maastrichtian). Sobs: richness observed.

A total of 23 species were recorded in fourteen fossiliferous strata ranging from the Lower Campanian to the Upper Maastrichtian in different localities in South America (Table 1 and Table S1). In relation to the total number of species, eight (34.78%) were restricted to the Campanian, five (21.73%) to the Maastrichtian, and 10 (43.47%) were distributed in both the Campanian and Maastrichtian (Table S1). The Chao1 richness estimator indicated a richness of 57 species during the Late Cretaceous, with a 95% confidence interval of 36 to 115 species (Fig. 1).

**Figure 2:**
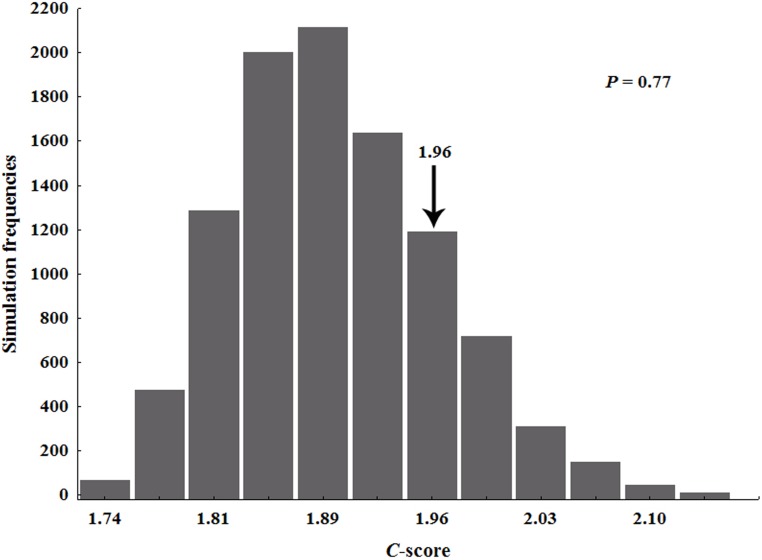
Frequency distribution of checkerboard *C*-scores obtained from 10,000 simulations produced by randomizations of titanosaurian assemblages. Arrow indicates the observed mean and *P* is the probability of the observed mean to be statistically greater than that expected.

Evaluations of the species encountered in each stratigraphic formation indicated that the Anacleto and Allen formations were the richest, with seven and six species distributed among the Campanian and Maastrichtian, respectively (Table 1 and Table S1). The analyses of species co-occurrence in fourteen stratigraphic formations indicated that all of the species formed checkerboard units in the presence-absence matrix. The observed *C-score* index was 1.96, which did not differ significantly from the mean expected by chance (1.91, Fig. 2). The result is consistent with the hypothesis that coexistence of South American Titanosauria species during the Late Cretaceous was not structured by deterministic processes.

**Figure 3:**
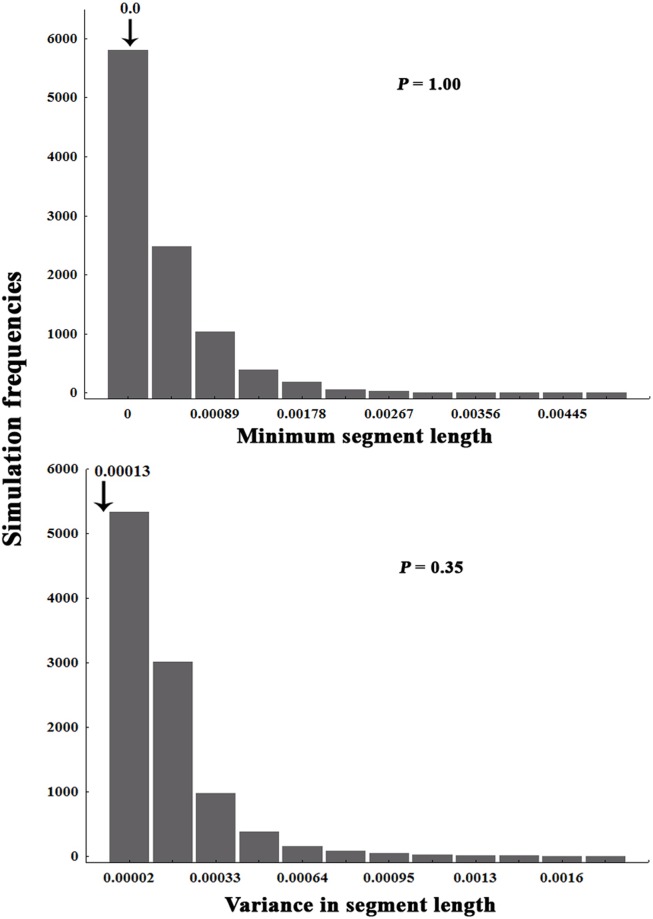
Observed and expected size overlap of Titanosauria species during the Late Cretaceous in South America. The dimensions used were minimum segment length and variance in segment length, with values log transformed. The arrows indicate observed means and *P* indicates the probability that the observed value is greater than the expected value (10,000 simulations).

The species themselves showed great variability with respect to their estimated sizes. *Rocasaurus muniozi* had the lowest estimated size (8 m long), whereas *Puertasaurus reuili* was estimated to be 30 m long [14], [37] (Table S1). Size-overlap analyses, based on minimum segment lengths, indicated that no mean overlap significantly larger than expected was observed in the titanosaurian assemblages (Fig. 3). The size-overlap analysis based on the variance of segment length showed that no mean overlap significantly smaller than random occurred in the titanosaurian assemblages (Fig. 3). Therefore, both analyses indicated a lack of structuring in the assemblages.

## Discussion

The fossil record points to the South American continent as having had a diverse assemblage of Titanosauria, and estimators of species richness indicate an even greater species richness during the Late Cretaceous (Early Campanian-Late Maastrichtian). This high species richness was possibly influenced by the availability and occupation of ecological niches left by the diplodocoids sauropods after their extinction in the Late Coniacian, resulting in a rich diversity of forms and sizes within the clade Titanosauria [10], [12]. Additionally, other ecological factors such as the association of titanosaurs with inland environments and a diet adapted to ingesting angiosperms may have contributed to clade Titanosauria diversification during this period [54].

The species richness could also have been related to the structural complexity of the habitats occupied by titanosaurian assemblages during the Late Cretaceous. Dinosaurs that coexisted during the Late Jurassic exhibit close associations with the characteristics of environments in which they lived, indicating the occurrence of structural patterns in those assemblages [55]. In modern ecosystems, structurally more complex habitats provided greater support for the maintenance of larger varieties of coexisting species as compared to more spatially homogeneous environments [55]–[56], and the richness and distributions of species that coexisted in time and space, when associated with environmental characteristics, can give rise to nonrandom patterns in ecological interactions and structured assemblages [25]–[26].

The results obtained by co-occurrence analyses of species richness demonstrated that the observed numbers of checkerboard units did not differ from random. This pattern is consistent with the hypothesis that the local coexistence of Titanosauria species during the Campanian and Maastrichtian in South America was not structured by ecological factors existing during the Late Cretaceous, such as resource limitations in the environment, interspecific competition, or predator-prey relationships.

The coexistence of species in an assemblage can be limited by ecological interactions known to be negative, such as interspecific competition for spatial and trophic niches, the occurrence of aggressiveness between individuals of the assemblage, species that develop specific preferences for certain types of habitats on a wide geographic scale, or predator-prey relationships [54]–[55], [57]–[61].

Random patterns in coexistence of Titanosauria species during the Late Cretaceous could have originated not through competitive interactions between species but through the influence of species showing specificity for a particular habitat type on a reduced spatial scale, distributions restricted to a particular period, endemism, or low abundances of some species [55], [57]–[58]. Additionally, the lack of structure in species assemblages can be driven by the stochastic nature of extinction events, and would be demonstrated by decreasing numbers of checkerboard units in the co-occurrence analyses [36].

Another important issue regarding the lack of structure in South American titanosaurian assemblages is the fact that species records in any particular formation can be influenced by factors such as taphonomic processes, the types of sedimentary paleoenvironment, and sampling efforts in fossil collecting. This latter aspect will be influenced by the numbers of paleontological explorations in each stratigraphic formation and by the environmental conditions at the sites where the fossiliferous strata are found (for example, sites located in forest areas that make fossil discovery more difficult in contrast to sites in arid environments with scarce vegetation cover that facilitates exploration).

Paleoecological studies should emphasize the importance of taphonomic processes for the different types of sedimentary paleoenvironments, since these factors can influence the fossil records of one or more species in stratigraphic formations. Diagenetic and biostratinomic processes, influenced by the nature of the sedimentary paleoenvironment at the site where the animal died (which can hinder fossilization) and the transport carcasses to different assemblages, will determine the number of specimens preserved in place and, consequently, estimated species richness [62]–[65] – but may also generate random patterns of species distributions. Thus, fossiliferous formations with low species richness or a set of under-sampled taxa could provide insufficient paleoecological data, making any structuring of assemblages of extinct species undetectable.

The analysis of size-overlapping in this study indicated a lack of structure in the Titanosauria assemblage, suggesting that the sizes of these dinosaurs were not a determinant factor for species coexistence in time and space. The random patterns attributed to body size overlapping among vertebrates in general may be due to local extinctions, non-limited food availability, or reduced population sizes [36]. Other factors such as genetic drift, invasion, or colonization by other species can also cause these patterns in terrestrial vertebrate assemblages [66].

Ecological differences between sauropod lineages could also have been associated with certain morphological attributes, such as body size and differences in dentition, shape of the necks and cranial morphology [7], [14], [67]–[68]. Morphological variations between species that coexisted in a given area can direct the utilization of certain types of resources, establishing guilds of morphologically similar species and determining the levels of overlap between them [61].

Competitive interactions for food resources and habitat utilization in bird assemblages can become reduced through morphometric variations related to the size and shape of the beak, the length of metatarsus, or body size [69]. The morphological differences between phylogenetically closely related species in lizard assemblages in Central America contributed to their segregation with respect to microhabitat utilization [70]. Thus, morphological variations will significantly contribute to niche segregation between species, making assemblage coexistence possible and determining how available resources in the environment will be utilized by each species [71].

Another important aspect that should be taken into consideration concerns possible historical effects on South American titanosaurian assemblages that occurred during the Late Cretaceous. The preference for, and utilization of, particular resources by Titanosauria lineages could have been strongly influenced by the evolutionary histories of the different clades. Phylogenetic effects include important processes that will determine the ecologies of large numbers of species (as opposed to putative interactions between members of the assemblage in terms of the utilization of available resources), reflecting the evolutionary histories of different lineages that diverged over millions of years [72]–[73]. Body size is strictly related to phylogenetic structures in different clades, suggesting that stabilizing selection processes may have been involved in the evolution of this character – which would be expected, as body size correlates with various ecological attributes and the life histories of the species [74].

## Conclusions

It is possible to conclude that the species richness of Titanosauria during the Late Cretaceous in South America was influenced by various ecological processes associated with the extinction events of various sauropods groups during this period and habitat structures that provided support for the maintenance of high species diversity in the assemblage. The observed patterns of co-occurrence and size overlapping suggest the existence of random processes and a lack of structuring in this assemblage. It is likely that these processes originated from phylogenetic inertia, associated with the occurrence of stabilizing selection, and that extinction events and historical factors had important roles in the formation of titanosaurian assemblages during the Late Cretaceous, more than did strictly ecological factors. Nonetheless, diagenetic and biostratinomic processes (influenced by the nature of the sedimentary paleoenvironment) can cause random species distribution patterns, making structuring of those undetectable.

## Supporting Information

Table S1 **Species of Titanosauria recorded in the stratigraphic formations of the Late Cretaceous in South America.** The number of recorded fossils (n) and whole information were obtained from the matrix of data available in the Paleobiology Database [1] and in the literature.
(DOC)
