# Supplementary material for: Species Richness and Evidence of Random Patterns in Assemblages of South American Titanosauria during the Late Cretaceous (Campanian–Maastrichtian)
Source: PLoS One. 2014 Sep 23;9(9):e108307. doi: 10.1371/journal.pone.0108307 (PMC4172772; doi:10.1371/journal.pone.0108307)
Supplement: Table S1 — Species of Titanosauria recorded in the stratigraphic formations of the Late Cretaceous in South America. The number of recorded fossils (n) and whole information were obtained from the matrix of data available in the Paleobiology Database [1] and in the literature. (DOC) [file pone.0108307.s001.doc]

**Supporting Information for**

**Species richness and evidence of random patterns in assemblages of South American Titanosauria during the Late Cretaceous (Campanian–Maastrichtian)**

**Washington Luiz Silva Vieira^1*^, Kleber Silva Vieira^1^, Rômulo Pantoja Nóbrega^1^, Paulo Fernandes Guedes Pereira Montenegro^1^, Gentil Alves Pereira Filho^2^, Gindomar Gomes Santana^3^, Rômulo Romeu Nóbrega Alves ^3, 4^, Waltécio de Oliveira Almeida^5^, Alexandre Vasconcellos^6^**

^1.^Laboratório de Ecofisiologia Animal, Departamento de Sistemática e Ecologia, Universidade Federal da Paraíba, CEP 58059-970 João Pessoa, PB, Brazil.

^2.^Museu de Zoologia da Universidade de São Paulo, Av. Nazaré, 481, CEP 04263-000, Ipiranga, São Paulo, SP, Brazil.

^3.^Programa de Pós-Graduação em Ecologia e Conservação (PPGEC)/Departamento de Biologia, Universidade Estadual da Paraíba, CEP 58109-753, Campina Grande, Paraíba, Brazil.

^4.^Departamento de Biologia, Universidade Estadual da Paraíba, CEP 58109-753, Campina Grande, Paraíba, Brazil.

^5.^Departamento de Química Biológica, Centro de Ciências Biológicas e da Saúde, Universidade Regional do Cariri – URCA, Campus do Pimenta, CEP 63105-000, Crato, CE, Brazil.

^6^. Departamento de Sistemática e Ecologia, Universidade Federal da Paraíba, CEP 58059-970 João Pessoa, PB, Brazil.

* E-mail: wlsvieira@yahoo.com.br

**Supplementary Table**

**Table S1. Species of Titanosauria recorded in the stratigraphic formations of the Late Cretaceous in South America**. The number of recorded fossils (n) and whole information were obtained from the matrix of data available in the Paleobiology Database [1] and in the literature.

| **Species** | **N** | **Overall length (m)** | **Time** | **Stratigraphy** | **Country** | **Reference Selected** |
| --- | --- | --- | --- | --- | --- | --- |
| *Adamantisaurus mezzalirai* Santucci and Bertini 2006 | 1 | 15 | Lower Campanian (83.5 Ma) | Adamantina Formation | Brazil | [1-6] |
| *Aeolosaurus colhuehuapiensis* Casal, Martinez, Luna, Sciutto, & Lamanna, 2007 | 1 | 15 | Lower Campanian to Late Maastrichtian (83.5 - 65.5 Ma) | Bajo Barreal Formation | Argentina | [1, 2, 6, 7] |
| *Aeolosaurus rionegrinus* Powell, 1987 | 2 | 15 | Lower Campanian to Late Maastrichtian (83.5 - 65.5 Ma) | Los Alamitos Formation,  Angostura Colorada Formation | Argentina | [1, 2, 4, 6, 8, 9] |
| *Antarctosaurus wichmanianus* Huene 1929 | 7 | 35 | Lower Campanian to Late Maastrichtian (83.5 - 65.5 Ma) | Plottier Formation,  Anacleto Formation,  Palacio Formation,  Adamantina Formation | Argentina,  Urugay and  Brazil | [1-4, 6, 10, 11] |
| *Argyrosaurus superbus* Lydekker, 1893 | 7 | 30 | Lower Campanian to Late Maastrichtian (83.5 - 65.5 Ma) | Bajo Barreal Formation | Argentina | [1, 2, 4, 6] |
| *Atacamatitan chilensis* Kellner, Rubilar-Rogers, Vargas & Suarez, 2011 | 1 | ? | Late Maastrichtian (65.5 Ma) | Tolar Formation | Chile | [12] |
| *Barrosasaurus casamiquelai* Salgado and Coria. 2009 | 1 | 15 | Lower to Middle Campanian (83.5 - 70.6 Ma) | Anacleto Formation | Argentina | [1, 2, 6, 13] |
| *Baurutitan britoi* Kellner, Bertini and Trotta, 2005 | 1 | 12 | Lower to Late Maastrichtian (70.6 - 65.5 Ma) | Marilia Formation | Brazil | [1, 2, 6, 14-17] |
| *Bonatitan reigi* Martinelli & Forasiepi, 2004 | 1 | 12 | Lower Campanian to Late Maastrichtian (83.5 - 65.5 Ma) | Allen Formation | Argentina | [1, 2, 6, 18] |
| *Bonitasaura salgadoi* Apesteguia, 2004 | 1 | 9 | Lower Campanian (83.5 Ma) | Bajo de la Carpa Formation | Argentina | [1, 2, 6, 19, 20] |
| *Gondwanatitan faustoi* Kellner & Azevedo, 1999 | 2 | 9 | Lower Campanian to Late Maastrichtian (83.5 - 65.5 Ma) | Cambambe Formation, Adamantina Formation | Brazil | [1, 2, 6, 14, 21] |
| *Laplatasaurus araukanicus* Huebe 1929 | 4 | 18 | Lower Campanian to Late Maastrichtian (83.5 - 65.5 Ma) | Anacleto Formation,  Allen Formation,  Palacio Formation | Argentina and  Uruguay | [1, 2, 4, 6, 22-24] |
| *Maxakalisaurus topai* Kellner, Campos, Azevedo, Trotta, Henriques, Craik, and Silva 2006 | 1 | 13 | Lower Campanian (83.5 Ma) | Adamantina Formation | Brazil | [1, 2, 6, 17, 25] |
| *Narambuenatitan palomoi* Filippi, Garcia, and Garrido, 2011 | 1 | ? | Lower to Middle Campanian (83.5 - 70.6 Ma) | Anacleto  Formation | Argentina | [26] |
| *Neuquensaurus australis* (Lydekker 1893) | 6 | 15 | Lower Campanian to Late Maastrichtian (83.5 - 65.5 Ma) | Anacleto Formation, Bajo de la Carpa Formation,  Allen Formation, Palacio Formation | Argentina and  Uruguay | [1, 2, 6, 27] |
| *Panamericansaurus schroederi* Calvo and Porfiri 2010 | 1 | 11 | Lower Campanian to Late Maastrichtian (83.5 - 65.5 Ma) | Allen Formation | Argentina | [1, 2, 6, 28] |
| *Pellegrinisaurus powelli* Salgado 1996 | 1 | 25 | Lower to middle Campanian (83.5 - 70.6 Ma) | Anacleto Formation | Argentina | [1, 2, 4, 6, 16, 25, 29] |
| *Pitekunsaurus macayai* Filipi and Garrido 2008 | 1 | 11 | Lower to middle Campanian (83.5 - 70.6 Ma) | Anacleto Formation | Argentina | [1, 2, 6, 30] |
| *Puertasaurus reuili* Novas, Salgado, Calvo, and Agnolin 2005 | 1 | 30 | Middle Campanian to Late Maastrichtian (70.6 - 65.5 Ma) | Pari Aike Formation | Argentina | [1, 6, 31, 32] |
| *Rocasaurus muniozi* Salgado and C. Azpilicueta. 2000 | 1 | 8 | Lower to Late Campanian (83.5 - 70.6 Ma) | Allen Formation | Argentina | [1, 2, 6, 24, 33] |
| *Saltasaurus loricatus* Bonaparte and powell 1980 | 1 | 12 | Lower to Late Maastrichtian (70.6 - 65.5 Ma) | Lecho Formation | Argentina | [1, 2, 6, 30, 34-36] |
| *Trigonosaurus pricei* Campos, Kellner, Bertini, and Santucci 2005 | 1 | 10 | Lower to Late Maastrichtian (70.6 - 65.5 Ma) | Marilia Formation | Brazil | [1, 2, 4, 16, 6] |
| *Uberabatitan ribeiroi* Salgado and Carvalho 2008 | 1 | 20 | Lower to Late Maastrichtian (70.6 - 65.5 Ma) | Marilia Formation | Brazil | [1, 2, 6, 37] |

**References**

1. Paleobiology Database (2000). Available: <http://www.paleodb.org/cgi-bin/bridge.pl>. Accessed 25 January 2013.
2. Peczkis J (1995) Implications for body-mass estimates for dinosaurs. J Vert Paleontol 14: 520-533.
3. Kellner AWA, Campos DA (2000) Brief review of dinosaur studies and perspectives in Brazil. An Acad Bras Ciênc 72(4): 509–538.
4. Powell JE (2003) Revision of South American titanosaurid dinosaurs: paleobiological, paleogeographical and phylogenetic aspects. Records Queen Vict Mus 111: 1-173
5. Santucci RM, Bertini RJ (2006) A new titanosaur from western São Paulo State, Upper Cretaceous Bauru Group, south-east Brazil. Palaeontology 49(1): 59–66.
6. Sander PM, Christian A, Clauss M, Fechner R, Gee CT, Griebeler EM, Gunga HC, Hummel J, Mallison H, Perry SF, Preuschoft H, Rauhut OWM, Remes K, Tütken T, Wings O, Witzel U (2011) Biology of the sauropod dinosaurs: the evolution of gigantism. Biol Rev 86: 117-155.
7. Casal G, Martínez RD, Luna M, Sciutto JC, Lamanna MC (2007) *Aeolosaurus colhuehuapensis* sp. nov. (Sauropoda, Titanosauria) de la Formación Bajo Barreal, Cretácico Superior de Argentina. Re. Bras Paleontol 10(1): 53–62.
8. Carpenter K, Alf K (1994) Global distribution of dinosaur eggs, nests, and babies. In: Carpenter K, Hirsch KF, Horner JR, editors. Dinosaur Eggs and Babies. Cambridge: Cambridge University Press. pp 15-30.
9. Lopes RP, Buchmann FSC (2008) Fossils of titanosaurs (Dinosauria, Sauropoda) from a new outcrop in Triângulo Mineiro, southeastern Brazil. Rev. Bras Paleontol 11(1): 69-72.
10. Bonaparte JF, Bossi G (1967) Sobre la presencia de dinosaurios en la Formación Pirgua del Grupo Salta y su significado cronológico. Acta Geol Lill 9: 25-44.
11. Candeiro CRA, Martinelli AG, Avilla LS, Rich TH (2006a) Tetrapods from the Upper Cretaceous (Turonian–Maastrichtian) Bauru Group of Brazil: a reappraisal. Cretaceous Res 27: 923–946.
12. Kellner AWA, Rubilar-Rogers D, Vargas A, Suarez M (2011) A new titanosaur sauropod from the Atacama Desert, Chile. An Acad Bras Ciênc 83(1): 211–219.
13. Salgado L, Coria RA (2009) *Barrosasaurus casamiquelai* gen. et sp. nov., a new titanosaur (Dinosauria, Sauropoda) from the Anacleto Formation (Late Cretaceous: early Campanian) of Sierra Barrosa (Neuquén, Argentina). Zootaxa 2222: 1–16.
14. Kellner AWA, Campos DA, Trotta MNF (2005) Description of a titanosaurid caudal series from the Bauru Group, Late Cretaceous of Brazil. Arq Mus Nac 63(3): 529-564.
15. Campos DA, Kellner AWA (1999) On some sauropod (Titanosauridae) pelves from the continental Cretaceous of Brazil. In: Tomida Y, Rich TH, Vickers-Rich P, editors. Proceedings of the Second Gondwanan Dinosaur Symposium. National Science: Museum Monographs 15. pp 143-166.
16. Campos DA, Kellner AWA, Bertini RJ, Santucci RM (2005) On a titanosaurid (Dinosauria, Sauropoda) vertebral column from the Bauru Group, Late Cretaceous of Brazil. Arq Mus Nac 63(3): 565–593.
17. Candeiro CRA, Santos AR, Rich TH, Marinho TS, Oliveira EC (2006b) Vertebrate fossils from the Adamantina Formation (Late Cretaceous), Prata paleontological district, Minas Gerais state, Brazil. Géobios 39: 319-327.
18. Martinelli A, Forasiepi AM (2004) Late Cretaceous vertebrates from Bajo de Santa Rosa (Allen Formation), Río Negro province, Argentina, with the description of a new sauropod dinosaur (Titanosauridae). Rev Mus Argen Cienc Nat 6(2): 257–305.
19. Apesteguía S (2004) *Bonitasaura salgadoi* gen. et sp. nov.: a beaked sauropod from the Late Cretaceous of Patagonia. Naturwissenschaften 91: 493-497.
20. Gallina PA, Apesteguía S (2011) Cranial anatomy and phylogenetic position of the titanosaurian sauropod *Bonitasaura salgadoi*. *Acta Palaeontol Pol* 56(1): 45–60.
21. Kellner AWA, Azevedo SAK (1999). A new sauropod dinosaur (Titanosauria) from the Late Cretaceous of Brazil. In: Tomida Y, Rich TH, Vickers-Rich P, editors. Proceedings of the Second Gondwanan Dinosaur Symposium. National Science: Museum Monographs 15. pp 111-142.
22. Bonaparte JF, Novas FE (1985) *Abelisaurus comahuensis*, n.g., n.sp., Carnosauria del Crétacico Tardio de Patagonia. Ameghiniana 21: 2-4.
23. Bonaparte JF (1996) Cretaceous tetrapods of Argentina. *Mün Geo Abhan* 30:73–130.
24. Salgado L, Azpilicueta C (2000) Un nuevo saltasaurino (Sauropoda, Titanosauridae) de la provincia de Río Negro (Formación Allen, Cretácico Superior), Patagonia, Argentina. Ameghiniana 37(3): 259-264.
25. Kellner AWA, Campos DA, Azevedo SAK, Trotta MNF, Henriques DDR, Craik MMT, Silva HP (2006) On a new titanosaur sauropod from the Bauru Group, Late Cretaceous of Brazil. Bol Mus Nac, Geol 74: 1-31.
26. Filippi LS, García RA, Garrido AC (2011) A new titanosaur sauropod dinosaur from the Upper Cretaceous of North Patagonia, Argentina. *Acta Palaeontol Pol* 56: 1-50.
27. Zaher H, Pol D, Carvalho AB, Nascimento PM, Riccomini C, Larson P, Juarez-Valieri R, Pires-Domingues R, Silva NJ, Campos DA (2011) A complete skull of an Early Cretaceous sauropod and the evolution of advanced titanosaurians. PLoS One 6(2): 1-10.
28. Calvo JO, Porfiri JD (2010) *Panamericansaurus schroederi* gen. nov. sp. nov. Un nuevo Sauropoda (Titanosauridae-Aeolosaurini) de la Provincia del Neuquén, Cretácico Superior de Patagonia, Argentina. Braz Geo J 1: 100-115.
29. Salgado L (1996) *Pellegrinisaurus powelli* nov. gen. et sp. (Sauropoda, Titanosauridae) from the Upper Cretaceous of Lago Pellegrini, northwestern Patagonia, Argentina. Ameghiniana 33(4): 355-365.
30. Filippi LS, Garrido AC (2008) *Pitekunsaurus macayai* gen. et sp. nov., nuevo titanosaurio (Saurischia, Sauropoda) del Cretácico Superior de la Cuenca Neuquina, Argentina. Ameghiniana 45(3): 575-590.
31. Novas FE, Salgado L, Calvo JO, Agnolín FL (2005) Giant titanosaur (Dinosauria, Sauropoda) from the Late Cretaceous of Patagonia. Rev Mus Argen Cienc 7(1): 37-41.
32. Gonzalez Riga BJ (2010) Paleobiology of South American titanosaurs. In: Calvo J, Porfiri J, Gonzalez Riga B, Dos Santos D, editors. Paleontología y dinosaurios desde America Latina, EDIUNC, Universidad Nacional de Cuyo. pp 125-141.
33. Salgado L, Coria RA (2005.) Sauropods of Patagonia: Systematic update and notes on global sauropod evolution. In. Tidwell, V, Carpenter K, editors. Thunder-Lizards: The Sauropodomorph Dinosaurs. Bloomington and Indianapolis: Indiana University Press. pp 430-453.
34. Bonaparte JF, Powell JE (1980) A continental assemblage of tetrapods from the Upper Cretaceous beds of El Brete, northwestern Argentina (Sauropoda-Coelurosauria-Carnosauria-Aves). Mem. Soc. Geol. de France 139: 19-28.
35. Upchurch P, Barrett PM, Dodson P (2004) Sauropoda. In: Weishampel DB, Dodson P, Osmólska H, editors. The Dinosauria, 2nd ed. Berkeley and Los Angeles: University of California Press. pp 259-322.
36. Wilson JA, Upchurch P (2003) A revision of Titanosaurus Lydekker (Dinosauria-Sauropoda), the first dinosaur genus with a 'Gondwanan' distribution. J Sys Palaeontol 1(3): 125-160.
37. Salgado L, Carvalho IS (2008) *Uberabatitan ribeiroi*, a new titanosaur from the Marília Formation (Bauru Group, upper Cretaceous), Minas Gerais, Brazil. Palaeontology, 51 (4): 881–901.
